# Tracking Official Development Assistance for Reproductive Health in Conflict-Affected Countries

**DOI:** 10.1371/journal.pmed.1000090

**Published:** 2009-06-09

**Authors:** Preeti Patel, Bayard Roberts, Samantha Guy, Louise Lee-Jones, Lesong Conteh

**Affiliations:** 1 King's College London, London, United Kingdom; 2 Conflict and Health Programme, London School of Hygiene & Tropical Medicine, London, United Kingdom; 3 RAISE Initiative, Marie Stopes International, London, United Kingdom; 4 Marie Stopes International, London, United Kingdom; 5 Swiss Centre for International Health, Swiss Tropical Institute, Basel, Switzerland; United Nations High Commissioner for Refugees (UNCHR), Switzerland

## Abstract

**Background:**

Reproductive health needs are particularly acute in countries affected by armed conflict. Reliable information on aid investment for reproductive health in these countries is essential for improving the efficiency and effectiveness of aid. The purpose of this study was to analyse official development assistance (ODA) for reproductive health activities in conflict-affected countries from 2003 to 2006.

**Methods and Findings:**

The Creditor Reporting System and the Financial Tracking System databases were the chosen data sources for the study. ODA disbursement for reproductive health activities to 18 conflict-affected countries was analysed for 2003, 2004, 2005, and 2006. An average of US$20.8 billion in total ODA was disbursed annually to the 18 conflict-affected countries between 2003 and 2006, of which US$509.3 million (2.4%) was allocated to reproductive health. This represents an annual average of US$1.30 disbursed per capita in the 18 sampled countries for reproductive health activities. Non-conflict-affected least-developed countries received 53.3% more ODA for reproductive health activities than conflict-affected least-developed countries, despite the latter generally having greater reproductive health needs. ODA disbursed for HIV/AIDS prevention and treatment increased by 119.4% from 2003 to 2006. The ODA disbursed for other direct reproductive health activities declined by 35.9% over the same period.

**Conclusions:**

This study provides evidence of inequity in disbursement of reproductive health ODA between conflict-affected countries and non-conflict-affected countries, and between different reproductive health activities. These findings and the study's recommendations seek to support initiatives to make aid financing more responsive to need in the context of armed conflict.

## Abstract 2

Preeti Patel and colleagues report inequity in the disbursement of official development assistance for reproductive health between countries affected by conflict and those unaffected.

## Editors' Summary

**Background:**

Reproductive health concerns the bodily functions and systems that are involved in conceiving and bearing offspring. A reproductively healthy person is able to have a responsible, satisfying and safe sex life and to reproduce if and when they chose to do so. More specifically, to ensure their reproductive health, both men and women need access to safe and effective birth control methods, they need to know how to avoid sexually transmitted diseases (including HIV/AIDS), and they need access to treatment should they become infected. Women also need access to appropriate health-care services to safeguard their own health and their offspring's health during pregnancy and childbirth. Reproductive health is essential for the wellbeing of individuals and families and for the social and economic development of nations. Consequently, some of the official development assistance (ODA) given to developing countries by wealthier nations and by international agencies is being used to improve reproductive health. Indeed, several of the Millennium Development Goals (internationally agreed targets designed to eradicate global poverty by 2015) are directly related to reproductive health, including the improvement of maternal health and the control of HIV/AIDS.

**Why Was This Study Done?:**

Many developing countries, such as Afghanistan, the Democratic Republic of Congo, Iraq, and Sudan, are experiencing violent conflicts. Such conflicts tend to slow down the development of low-income countries, and can also cause harm to reproductive health by damaging the health-service infrastructure and by increasing exposure to sexual violence. Although conflict-affected low-income countries rely heavily on international and humanitarian aid for basic health-care provision, there is little reliable information about how much of this aid is invested in reproductive health in such countries. This information is needed to ensure that development aid is used effectively. In this study, therefore, the researchers analyze the amount of ODA disbursed (the amount of official development money paid to recipient countries) for reproductive health activities in conflict-affected countries between 2003 and 2006.

**What Did the Researchers Do and Find?:**

The researchers identified eighteen countries (mostly “least-developed” countries as defined by the Organisation for Economic Co-operation and Development; OEDC) that had been at war at sometime during the study period. They obtained information on ODA disbursements for reproductive health activities mainly from the Creditor Reporting System (CRS) database, which is maintained by the OECD, but also from the Financial Tracking System (FTS) database, which is maintained by the United Nations Office for the Coordination of Humanitarian Affairs. An average of US$20.8 billion in ODA was disbursed annually to the 18 conflict-affected countries between 2003 and 2006. Only US$509.3 million (2.4%) of this was allocated to reproductive health. Put another way, each person living in these conflict-affected countries received US$1.30 per year for their reproductive health needs. By contrast, people in non-conflict-affected least-developed countries each received 50% more ODA for reproductive health activities, even though these countries often had better reproductive health indicators than the conflict-affected countries. The researchers also found that nearly half of ODA disbursed for reproductive health was used for HIV/AIDS-related activities. This portion of ODA increased slightly during the study period in the conflict-affected countries whereas ODA disbursed for other reproductive health activities fell by a third.

**What Do These Findings Mean?:**

Although these findings do not take into account money provided to conflict-affected developing countries for reproductive health activities by large philanthropic organizations, they nevertheless reveal an inequality between conflict-affected and non-conflict affected countries in terms of the development money provided for reproductive health. This is a worrying finding given that reproductive health tends to suffer in countries affected by war and poor reproductive health can slow down development. The findings of this study also suggest that funding for non-HIV reproductive health activities is declining in conflict-affected countries. Importantly, they also highlight additional research that is needed to ensure that donors of development aid can be more responsive in future to the reproductive health needs of conflict-affected countries.

**Additional Information:**

Please access these Web sites via the online version of this summary at http://dx.doi.org/10.1371/journal.pmed.1000090.

- This study is further discussed in a *PLoS Medicine* Perspective by Paul Spiegel and colleagues
- The World Health organization provides information about reproductive health, including information on its 2004 global strategy for reproductive health (in several languages)
- The US Centers for Disease Control and Prevention also provides information on reproductive health (in English and Spanish)
- Wikipedia has a page on reproductive health (note that Wikipedia is a free online encyclopedia that anyone can edit; available in several languages
- The United Nations provides information on the Millennium Development Goals
- The Organisation for Economic Cooperation and Development provides information on ODA through its Creditor Reporting System database; the United Nations Office for the Coordination of Humanitarian Affairs provides similar information for other donors through its Financial Tracking System database
- The Reproductive Health Response in Conflict Consortium promotes access to reproductive health programs in emergencies and advocates for policies that support reproductive health of persons affected by armed conflict

## Introduction

Reproductive health is fundamental to individuals, couples, and families, and the social and economic development of communities and nations [1]. Three of the eight Millennium Development Goals (MDGs; http://www.un.org/millenniumgoals/)—improving maternal health, reducing child mortality, and combating HIV/AIDS, malaria and other diseases—are directly related to reproductive and sexual health. Four other MDGs—eradicating poverty and hunger, achieving universal primary education, promoting gender equality and empowering women, and ensuring environmental sustainability—have a close relationship with reproductive health [2]–[4].

Studies suggest that funding for sexual and reproductive health programmes has consistently fallen short of the financial targets agreed to at the International Conference on Population and Development in 1994 [5]–[7]. However, further information is required on international investment patterns for reproductive health activities [3],[6],[8]. In particular, information on aid investment for reproductive health in conflict-affected countries is extremely limited, and no studies could be identified that tracked official development assistance (ODA) for reproductive health in conflict-affected countries. See Box 1 for definitions of these key terms.

**Box 1. Definitions of Key Terms**

**Reproductive health** follows the definition given in the International Conference on Population and Development in 1994. It refers to the constellation of methods, techniques, and services that contribute to reproductive health and well-being by preventing and solving reproductive health problems [54]. International guidelines on reproductive health in conflict-affected situations include reproductive activities on family planning, HIV/AIDS and sexually transmitted diseases, maternal and newborn health, and sexual and gender-based violence [55].

**Official development assistance, or ODA,** is defined as flows of official financing administered with the promotion of the economic development and welfare of developing countries as the main objective, including humanitarian aid [23],[56].

**Conflict-affected countries** were selected as having been at “war” at a point in the period 2000 to 2006 based upon the Uppsala University Conflict Database, with additional information used from the World Bank [38],[57]. As the conflicts could have finished during this 5 year period, the conflict-affected countries in the study sample included countries that were either at war or in a postwar phase. War is defined as major armed conflict in which there are over a 1,000 battle-related deaths in 1 year [58].

Most conflict-affected countries rely heavily upon international aid and humanitarian assistance for basic service provision as internal state capacities are limited [9]. Studies have shown the need for more international aid for conflict-affected countries [10]. Reliable information on the distribution of international aid to conflict-affected countries is essential to increase understanding of aid volumes, the efficiency and effectiveness by which aid is allocated, and accountability of both donor and recipient countries [11],[12].

Conflict-affected low-income countries have worse development indicators than non-conflict-affected low-income countries [9],[13]. Conflict can increase vulnerability to poor reproductive health as the health service infrastructure and human resources can be severely depleted; access to reproductive health services, information, and supplies reduced; exposure to sexual violence increased; and impoverishment and related risk-taking behaviour increased [14],[15]. Studies also indicate high demand and unmet need for reproductive health services among people affected by conflict [16],[17]. Despite this need, reproductive health has historically received insufficient attention in conflict-affected countries [18].

The purpose of this study was to analyse ODA disbursed for reproductive health activities in conflict-affected countries in 2003, 2004, 2005, and 2006. The specific objectives were: (i) to measure the absolute amount of ODA disbursed for reproductive health related activities to conflict-affected countries; (ii) to analyse the disbursement of reproductive health ODA between conflict-affected countries; (iii) to compare reproductive health ODA disbursed to conflict-affected countries and non-conflict-affected countries within the same income category; (iv) to analyse disbursement patterns of reproductive health ODA across different reproductive health-related activities; (v) to analyse disbursement patterns of reproductive health ODA across donors. This paper forms the basis of a long-term study to analyse trends over time of ODA for reproductive health in conflict-affected countries.

## Methods

### Data Source, Donors, and Recipient Countries

A literature review and key-informant interviews with representatives from academia, donor agencies, and nongovernmental organisations involved in global and national tracking studies were initially undertaken to help develop the study methodology. These confirmed that the most reliable and comprehensive data source was the Creditor Reporting System (CRS). The CRS is a publicly accessible, Web-based database on aid activities, developed and maintained by the Development Assistance Committee (DAC) of the Organisation of Economic Cooperation and Development (OECD) [19]. The CRS covers approximately 90% of all ODA to developing countries including conflict and postconflict-affected countries, and it includes humanitarian aid. The use of CRS for health tracking studies has also been justified in other published studies [20],[21].

All donors contributing to the CRS were included in this study. These included 22 DAC countries and 16 multilateral donors. The multilateral agencies include the UN agencies disbursing ODA, and major agencies such as World Bank and the Global Fund to fight AIDS, Tuberculosis and Malaria. These bilateral and multilateral donors account for over 90% of ODA including humanitarian assistance to least-developed countries [22]. Both bilateral and multilateral aid are included in the CRS and separately recorded to avoid double counting.

This study analysed only ODA disbursements rather than commitments, as commitments might not be allocated as pledged. Disbursements refer to the placement of resources at the disposal of a recipient country or agency, not the actual expenditure within the recipient country [23]. The disbursements were calculated as gross disbursements of constant US dollars with 2006 as the base year, so as to take into account inflation and donor exchange rate variations over the years analysed in the study. Deflator rates used by CRS were applied to incorporate exchange rate differences and inflation during the years in question [24].

The CRS includes the main funding approaches in which ODA is disbursed to conflict-affected and postconflict recipient countries. These include humanitarian aid; longer-term developmental programmatic or project funding; pooled funding such as common humanitarian funds for recipient countries, Sector-Wide Approaches (SWAps) and Basic Packages of Health Services; and general budget support in which donors provide aid to the recipient government without specifying the sectors where this aid should be allocated [25],[26].

While CRS includes humanitarian ODA [27], four relevant United Nations (UN) agencies do not report to CRS disbursements of their own institutional funds. These four UN agencies are the United Nations High Commissioner for Refugees (UNHCR), the United Nations Office for the coordination of Humanitarian Affairs (UNOCHA), the World Food Programme (WFP), and the World Health Organisation (WHO). These institutional funds are separate from the ODA these four agencies receive from bilateral and multilateral donor agencies for country-specific activities that are reported to CRS by the bilateral and multilateral donor agencies. To include the institutional funds of these four agencies in the study, we conducted an analysis of the disbursements by these four UN agencies using the Financial Tracking System (FTS), which specifically tracks humanitarian aid and is maintained by UNOCHA [28].

The focus of this study is ODA, and so the study does not include aid from large private philanthropic organisations, such as the Bill and Melinda Gates Foundation. Data from philanthropic organisations are not reported to CRS. This kind of aid is also often not comprehensive or standardised, and sufficiently reliable data on disbursements to conflict-affected countries could not be found to include in the study [29].

No ethical approval was required for the study as all the data used are in the public domain.

### Data Analysis

This study analysed data for 2003, 2004, 2005, and 2006. The time frame for analysis was chosen so as to provide data on recent disbursement patterns for reproductive health and so be prescient for those engaged in contemporary policy decision-making. Data from 2006 were the most recent data available at the time of the study. The 18 countries selected for the study were those that met the definition of conflict-affected (Box 1). All ODA data for each recipient country selected for the study were downloaded from the CRS and FTS database and analysed in an Excel-based database. The data analysis was cross-checked by study authors to ensure accuracy.

For the CRS analysis, CRS-labelled aid activities were selected that contributed either directly or indirectly toward reproductive health (Table 1). 100% of ODA disbursements for direct reproductive health activities were included in the analysis. Activities included in the analysis that were indirectly related to reproductive health were education; nutrition; general health; general budget support; humanitarian material relief assistance and services; and reconstruction relief and rehabilitation. A proportion of ODA disbursements from these indirect activities was allocated for inclusion in the analysis (Table 1).

**Table 1:** Creditor Reporting System activities included in analysis.

Activities^a^	Percent Allocation	Basis for Allocation
**Direct activities** b		Estimates based on calculations by NIDI and developed in the OECD 54th meeting of the Working Party on Statistics, June 2005 [59].
Population policy & admin. Management	100	
Reproductive health care^c^	100	
Family planning	100	
Personnel development for population & RH	100	
Social mitigation of HIV/AIDS	100	
HIV/AIDS and STD control	100	
**Indirect activities**		Estimates based on calculations by NIDI and developed in the OECD 54th meeting of the Working Party on Statistics, June 2005 [59].
Primary education	10	
Basic skills for youth and education	10	
Early childhood education	10	
Secondary education	10	
Health policy & admin. Management	10	
Basic health care	25	
Basic health infrastructure	25	
Basic nutrition	75	
Health education	25	
Health personnel development	25	
General budget support	2.11	Estimate based on average government expenditure on health for the 18 sampled countries (8.42%) [60].25% of this 8.42% was then allocated for RH based on NIDI estimates (see above).
Material relief assistance and services	1.94	Estimate based upon calculation of 7.76% of humanitarian ODA being allocated for the health sector using FTS data for 2003, 2004, 2005, 2006 [28]. 25% of this 7.76% was then allocated for RH based upon estimates calculated by NIDI estimates (see above).
Reconstruction relief and rehabilitation	1.94	As above.

a Activities are same as CRS purpose codes.

b Direct RH categories based on categories defined in the 1994 International Conference on Population and Development and subsequently used by van Dalen [50].

c Reproductive health care includes promotion of reproductive health; prenatal and postnatal care including delivery; prevention and treatment of infertility; prevention and management of consequences of abortion; safe motherhood activities.

Abbreviations: RH, Reproductive Health; NIDI, The Netherlands Interdisciplinary Demographic Institute.

Each of the CRS-labelled aid activities is accompanied in the CRS database by a numeric “purpose-code” that was used for the data analysis in the excel spreadsheet. An alternative method of analysis could have been to review and code the narrative text descriptions of each ODA project record, as used in other tracking studies [20],[21]. However, this was not required for this study, as the CRS categories/purpose codes for reproductive health are already defined and provide sufficient specificity for meaningful analysis. In addition, previous tracking studies on reproductive health-related activities showed that searching activities based on text descriptions was not considered reliable because the description of the activities to be funded was often absent or unspecific [30].

For the FTS analysis of institutional disbursements by UNHCR, UNOCHA, WFP, and WHO only, we searched the FTS database for paid contributions (including un-earmarked funds allocated by the four agencies) relating to the activities outlined in Table 1 for the 18 selected countries selected from 2003 to 2006. Deflator rates were applied as above, and proportions were allocated for reproductive health activities based upon those given in Table 1. The FTS does not provide data as detailed as CRS, and the FTS data were generally labelled under the categories of basic health care; nutrition; and humanitarian material relief assistance and services. The FTS data were then combined with the CRS data.

A comparative analysis of the combined study data was also made with ODA disbursed to nonconflict-affected countries, using CRS and FTS (for UNHCR, UNOCHA, WFP, and WHO only) data for nonconflict-affected countries. Of the 18 conflict-affected countries, only three were not in the OECD/DAC category of least-developed countries: Colombia, Iraq, and Sri Lanka. The 15 conflict-affected countries which were in the OECD/DAC category of least-developed countries were compared with the remaining 36 nonconflict-affected countries in the least-developed country category [31]. The procedures described above for analysing the data were used for the 36 nonconflict-affected countries.

## Results

The study analysis covered records from CRS and FTS for the 18 conflict-affected countries for 2003, 2004, 2005, and 2006. The average annual ODA disbursed for reproductive health to all the 18 countries during the 4 years was US$509.3 million (ranging from US$354.2 million in 2003 to US$643.1 million in 2005). This represented 2.4% of the US$20.8 billion average annual disbursement for all ODA to all the 18 countries during the review period (Table 2). This ODA for reproductive health was equivalent to an average of US$1.30 disbursed per capita per year for reproductive health in the conflict-affected countries. Table 3 shows the countries receiving the highest annual average per capita disbursement of reproductive health ODA between 2003 and 2006 were Uganda (US$4.80), Timor Leste (US$3.20), and Central African Republic (US$2.90). The countries with the lowest annual average per capita disbursement of reproductive health ODA were Colombia (US$0.10), Sri Lanka (US$0.30), and Myanmar (US$0.30).

**Table 2:** ODA disbursement in conflict-affected countries.

Country	RH ODA (USD Million)					All ODA (USD Million)					Annual Average ODA Per Capita (USD)		RH Percentage of All ODA
	RH ODA 2003	RH ODA 2004	RH ODA 2005	RH ODA 2006	Mean RH ODA 2003–6	All ODA 2003	All ODA 2004	All ODA 2005	All ODA 2006	Mean all ODA 2003–6	RH ODA	All ODA	
Afghanistan	34.5	43.6	48	56.1	45.5	1,320.3	1,534.4	2,361.4	2,544	1,940	1.50	64.90	2.3
Angola	29.9	20.6	32.6	32.0	28.8	511.9	1,158.2	416.6	241.3	582.0	1.80	36.60	4.9
Burundi	4.4	19.0	30.9	18.7	18.3	179.9	394.2	322.7	280.1	294.2	2.40	39.20	6.2
Central African Republic	12.8	10.0	12.8	11.0	11.7	87.5	113.1	107.8	102.4	102.7	2.90	25.70	11.4
Chad	9.3	15.5	12.4	16.0	13.3	186.3	248.5	307.6	230.7	243.3	1.40	25.10	5.5
Colombia^a^	2.2	2.8	5.2	8.2	4.6	900.2	555.8	717.5	1,005.2	794.7	0.10	17.40	0.6
Democratic Republic of Congo	45.7	33.1	48.0	54.7	45.3	7,591.8	1,567.2	1,502.1	1,780.0	3,110.3	0.80	54.10	1.5
Eritrea	12.7	15.0	11.1	9.0	12.0	228.6	171.8	256.5	83.3	185.0	2.70	42.10	6.5
Iraq^a^	18.0	33.2	122.9	84.7	64.7	2,377.7	3,407.7	23,918.7	8,424.9	9,532.2	2.20	331.00	0.7
Liberia	4.2	7.2	7.5	7.8	6.7	110.1	203.6	212.2	283.5	202.4	2.00	61.30	3.3
Myanmar	18.2	16.3	23.5	8.9	16.7	109.5	98.3	115.7	115.7	109.8	0.30	2.20	15.2
Nepal	37.0	36.8	35.9	57.4	41.7	367.7	306.8	339.7	369.8	346.0	1.50	12.80	12.1
Sierra Leone	6.9	12.7	18.6	8.1	11.6	288.8	246.6	245.8	261.3	260.6	2.10	47.40	4.5
Somalia	3.5	3.5	13.7	13.1	8.5	177.7	172.8	180.9	366.2	224.4	1.00	27.40	3.8
Sri Lanka^a^	4.2	4.5	9.1	4.8	5.7	458.4	488.1	855.9	757.0	639.9	0.30	30.90	0.9
Sudan	11.6	15.2	45.6	55.2	31.9	451.6	800.3	1,673.8	1,844.7	1,192.6	0.90	32.90	2.7
Timor Leste	2.6	1.5	2.4	5.1	2.9	176.0	148.3	182.6	208.7	178.9	3.20	198.80	1.6
Uganda	96.5	119.3	162.9	179.3	139.5	762.5	739.2	808.0	1,112.4	855.5	4.80	29.70	16.3
**All conflict total** b	**354.2**	**409.8**	**643.1**	**630.1**	**509.3**	**16,286.5**	**12,354.9**	**34,525.5**	**20,011.2**	**20,794.5**	**1.30**	**54.10**	**2.4**
LDC conflict total^c^	329.7	369.3	506.0	532.3	434.3	12,550.0	7,903.4	9,033.6	9,824.0	9,827.8	1.50	34.00	4.4
Non-conflict LDCs^d^	805.9	1,025.2	1,325.0	1,259.4	1,103.9	10,217.0	13,454.3	13,370.1	12,375.2	12,354.2	2.30	25.60	8.9
All LDCs	1,135.6	1,394.5	1,831.1	1,791.7	1,538.2	22,767.1	21,357.7	22,403.7	22,199.2	22,181.9	2.00	28.70	6.9

All data are in constant US$ with 2006 as the base year, using deflator rates used by CRS to incorporate donor exchange rate differences and inflation during the period in question.

a Non-LDC conflict-affected countries.

b Total for all 18 conflict-affected countries (both LDC and non LDC).

c Total for the 15 conflict-affected countries in the LDC category.

d Total for 36 non-conflict-affected countries in the LDC category.

Abbreviations: GDP, gross domestic product; LDC, least-developed countries; NA; not available; RH, reproductive health; USD, US dollars.

**Table 3:** Conflict-affected country indicators in 2005.

Country	HIV/AIDS Rate (%)^d^	Maternal Mortality Ratio^e^	Contraceptive Prevalence Rate^f^	Total Fertility Rate^g^	Total Population^g^	GDP per Capita (USD)^h^	Annual Average RH ODA per Capita (USD)
Afghanistan	<0.1	1,800	3.6	7.3	29.9	300	1.50
Angola	3.7	1,400	4.5	6.6	15.9	212	1.80
Burundi	3.3	1,100	10.0	6.8	7.5	107	2.40
Central African Republic	10.7	980	6.9	4.8	4	335	2.90
Chad	3.5	1,500	2.1	6.7	9.7	654	1.40
Colombia^a^	0.6	130	64.0	2.5	45.6	2,656	0.10
Democratic Republic of Congo	3.2	1,100	4.4	6.7	57.5	NA	0.80
Eritrea	2.4	450	5.1	5.3	4.4	209	2.70
Iraq^a^	<0.2	300	10.4	4.5	28.8	1,700	2.20
Liberia	3.5	1,200	5.5	6.8	3.3	161	2.00
Myanmar	1.3	380	32.8	2.3	50.5	219	0.30
Nepal	0.5	830	35.4	3.5	27.1	322	1.50
Sierra Leone	1.6	2,100	3.9	6.5	5.5	223	2.10
Somalia	0.9	1,400	NA	6.2	8.2	NA	1.00
Sri Lanka^a^	<0.1	58	49.6	1.9	20.7	1,200	0.30
Sudan	1.6	450	6.9	4.2	36.2	820	0.90
Timor Leste	<0.2	380	8.6	7.5	0.90	352	3.20
Uganda	6.7	550	18.2	7.1	28.8	303	4.80
**All conflict average** b	**2.5**	**894.9**	**16.0**	**5.4**	**21.4**	**730.6**	**1.30**
**LDC conflict average** c	**2.9**	**1,041.3**	**10.6**	**5.9**	**19.3**	**471.8**	**1.50**

2005 data used to provide approximate midpoint data for the period in question.

a Non-LDC conflict-affected countries.

b Average of all 18 conflict-affected countries (both LDC and non LDC).

c Average of the 15 conflict-affected countries in the LDC category.

d Adult (age 15–49) % rate HIV/AIDS for 2005 [61].

e Maternal deaths per 100,000 live births [62].

f Contraceptive prevalence rate for modern contraceptive methods only [63].

g Data from United Nations Population Fund [64].

h Data from the International Monetary Fund [65].

Abbreviations: GDP, gross domestic product; LDC, least-developed countries; NA, not available; RH, reproductive health; USD, US dollars.

Table 3 provides key reproductive health, demographic, and economic data for all the 18 sampled countries. The table illustrates inequity between the 18 sampled countries in annual average per capita ODA disbursed for reproductive health when compared to health outcomes and per capita GDP. For example, Timor Leste (US$3.20) and Iraq (US$2.20), which had better reproductive health indicators, received more per capita ODA than countries with worse reproductive health indicators such as Somalia (US$1.00) and Democratic Republic of Congo (US$0.80).

The study also compared ODA disbursed for reproductive health for 15 of the 18 conflict-affected countries which were classified as “least-developed countries” by OECD/DAC with the remaining 36 nonconflict-affected countries in the OECD/DAC “least-developed countries” classification [31]. The average annual per capita ODA disbursed for reproductive health from 2003 to 2006 to the nonconflict-affected least-developed countries was US$2.30. This was 53.3% higher than the US$1.50 in reproductive health disbursed per capita for the 15 conflict-affected least-developed countries (Table 2). The amount is higher for nonconflict-affected least-developed countries despite the fact that the conflict-affected least-developed countries appear to have generally worse reproductive health indicators (with the notable exception of HIV/AIDS), while also having significantly lower GDP than the nonconflict-affected least-developed countries (Tables 3 and 4). The lower prioritisation of ODA for reproductive health in conflict-affected least-developed countries is highlighted by the fact that 4% of all ODA disbursed to conflict-affected least-developed countries was for reproductive health activities, compared to 9% in nonconflict-affected least-developed countries (Table 2).

**Table 4:** Least-developed country indicators 2005 (nonconflict-affected).

Country	HIV/AIDS Rate %^a^	Maternal Mortality Ratio^b^	Contraceptive Prevalence Rate^c^	Total Fertility Rate^d^	Total Population^d^	GDP per Capita 2005 (USD)^e^
Bangladesh	<0.1	570	47.3	3.1	141.8	400
Benin	1.8	840	7.2	5.6	8.4	592
Bhutan	<0.1	440	18.8	4.1	2.2	1,126
Burkina Faso	2.0	700	8.6	6.5	13.2	429
Cambodia	1.6	540	18.5	3.9	14.1	430
Cape Verde	0.1	210	46.0	3.6	0.50	2,065
Comoros	<0.1	400	19.3	4.6	0.80	614
Djibouti	3.1	650	NA	4.8	0.80	973
Equatorial Guinea	3.2	680	NA	5.9	0.50	6,205
Ethiopia	2.1	720	6.3	5.7	77.4	153
Gambia	2.4	690	8.9	4.5	1.5	305
Guinea	1.5	910	4.2	5.7	9.4	354
Guinea Bissau	3.8	1,100	3.6	7.1	1.6	190
Haiti	3.8	670	22.3	3.8	8.5	478
Kiribati	NA	NA	NA	NA	0.1	672
Laos	0.1	660	28.9	4.6	5.9	485
Lesotho	23.2	960	29.5	3.5	1.8	620
Madagascar	0.5	510	16.7	5.2	18.6	281
Malawi	14.1	1,100	26.1	5.9	12.9	161
Maldives	0.2	120	33.0	4.1	0.3	2,349
Mali	1.7	970	5.7	6.8	13.5	431
Mauritania	0.7	820	5.1	5.6	3.1	662
Mozambique	16.1	520	11.8	5.3	19.8	331
Niger	1.1	1,800	4.3	7.7	14	273
Rwanda	3.1	1,300	4.3	5.5	9	242
Samoa	NA	130	NA	4.2	0.20	1,832
Sao Tome & Principe	NA	NA	27.4	NA	0.20	430
Senegal	0.9	980	8.2	4.8	11.7	738
Solomon Islands	NA	220	NA	4.1	0.5	611
Tanzania	6.5	950	16.9	4.7	38.3	336
Togo	3.2	510	9.3	5.1	6.1	376
Tuvalu	NA	NA	NA	NA	0.01	NA
Vanuatu	NA	130*	NA	3.9	0.20	1,530
Yemen	<0.2	430	9.8	5.9	21	585
Zambia	17.0	830	22.6	5.4	11.7	626
Zimbabwe	20.1	1,100	50.4	3.4	13	382
**Non-conflict LDC average**	**4.5**	**719.69**	**18.0**	**5.0**	**13.4**	**808**

2005 data used to provide approximate midpoint data for the period in question.

a Adult (age 15–49) % rate HIV/AIDS for 2005 [61].

b Maternal deaths per 100,000 live births [62].

c Contraceptive prevalence rate for modern contraceptive methods only [63].

d Data from United Nations Population Fund [64].

e Data from the International Monetary Fund [65].

Abbreviations: GDP, gross domestic product; LDC, least-developed countries; NA, not available; USD, US dollars.

The activities to which the reproductive health-related ODA to conflict-affected countries was disbursed are given in Table 5. This table shows that an annual average of US$237.67 million was disbursed for direct HIV/AIDS activities (“HIV/AIDS and STD control” and “Social mitigation of HIV/AIDS”). This amount represents almost half (46.7%) of $509.29 million in ODA annually disbursed for reproductive health (direct and indirect). The next highest activities were for basic health care (10.7%) and basic health infrastructure (8.9%). The average annual ODA disbursed for direct reproductive health activities, excluding direct HIV/AIDS activities, was $70.45 million, or 13.8% of the average annual ODA disbursed for all reproductive health activities.

**Table 5:** Distribution of reproductive health ODA to conflict-affected countries, by activity (US$ million).

Category	RH ODA 2003	RH ODA 2004	RH ODA 2005	RH ODA 2006	Total RH ODA	Annual Average RH ODA	Percentage of All RH ODA
**Direct activities**							
Population policy and administrative management	44.37	39.95	13.35	26.00	123.67	30.92	6.07
Reproductive health care	43.28	12.58	31.87	32.40	120.13	30.03	5.90
Family planning	10.51	20.10	2.43	1.90	34.94	8.74	1.72
Personnel development for population and RH	0.00	0.02	0.42	2.60	3.04	0.76	0.15
Social mitigation of HIV/AIDS	0.00	0.11	1.04	6.90	8.05	2.01	0.39
HIV/AIDS and STD control	139.14	198.72	306.38	298.40	942.64	235.66	46.27
**Indirect activities**							
Primary education	12.63	19.55	27.35	16.36	75.89	18.97	3.72
Nonformal education	1.60	0.92	1.88	1.31	5.71	1.43	0.28
Preschool education (0 up to 8 years)	0.18	0.22	0.27	0.56	1.23	0.31	0.06
Secondary education	0.57	0.48	1.10	1.18	3.33	0.83	0.16
Health policy and administrative management	5.23	4.98	7.97	6.97	25.15	6.29	1.24
Basic health care	35.14	47.06	53.37	82.58	218.15	54.54	10.71
Basic health infrastructure	10.67	8.27	105.55	57.58	182.07	45.52	8.94
Nutrition	12.04	9.34	34.36	41.70	97.44	24.36	4.78
Health education	1.44	1.81	0.95	0.45	4.65	1.16	0.23
Health personnel development	0.00	0.00	0.00	1.30	1.30	0.33	0.06
General budget support	5.83	5.63	7.27	10.09	28.82	7.21	1.42
Humanitarian: material relief assistance and services	28.31	34.37	38.31	32.99	133.98	33.50	6.58
Humanitarian: reconstruction, relief and rehabilitation	3.23	5.70	9.25	8.78	26.96	6.74	1.32
**Total RH ODA**	**354.17**	**409.81**	**643.12**	**630.05**	**2037.15**	**509.29**	**100.00**

Data for all 18 sampled conflict-affected countries (both least-developed and non-least-developed).

Abbreviations: RH, reproductive health; STD, sexually transmitted disease.

There was a substantial increase (77.9%) in funding for reproductive health activities to the 18 sampled countries between 2003 and 2006. This compares with a 22.9% overall increase in all ODA distributed to the 18 sampled countries. Data in Table 5 show that this growth in reproductive health ODA was largely due to a 119.4% increase in funding for HIV/AIDS activities over the 4 years, with HIV/AIDS activities accounting for 46.7% of all reproductive health ODA over the 4 years. The ODA disbursed for the other direct reproductive health activities declined by 35.9% over the same period. ODA for family planning fluctuated from US$10.51 million in 2003 to US$20.10 million in 2004 and down to US$1.90 million in 2006. ODA for reproductive health care also fluctuated from US$43.28 million in 2003 to US$12.58 million in 2004 and US$32.40 million in 2006.

The study also investigated the sources of ODA for reproductive health (Table 6). The donors disbursing the highest amount of absolute bilateral reproductive health related ODA were the United States, Japan, and the United Kingdom. Nearly half (41.94%) of the ODA disbursed for HIV/AIDS activities came from the United States, which disbursed an annual average of US$102.2 million for HIV/AIDS-prevention and -treatment activities between 2003 and 2006 to the sampled countries. The bilateral donors disbursing the highest proportion of their ODA to reproductive health were Ireland (5.8%), Denmark (5.3%), and Finland (4.2%). The total reproductive health ODA reported by UNHCR, UNOCHA, WFP, and WHO (using FTS) was US$62.91 million, or 3.1% of all the reproductive health ODA disbursed (Table 6).

**Table 6:** Donor disbursement of reproductive health ODA for sampled conflict-affected countries (US$ million).

Donor	RH ODA 2003	RH ODA 2004	RH ODA 2005	RH ODA 2006	RH ODA Annual Average 2003–6	Percent of All Donors RH ODA	All ODA Annual Average 2003–6	RH as Percent of All ODA
**Bilateral**								
Australia	3.42	3.66	0.56	2.75	2.60	0.51	203.92	1.28
Austria	0.15	0.39	0.27	0.20	0.25	0.05	301.33	0.08
Belgium	4.29	6.85	7.79	12.85	7.95	1.56	531.93	1.49
Canada	3.43	4.17	2.40	2.79	3.20	0.63	342.33	0.93
Denmark	4.30	4.46	6.40	5.72	5.22	1.02	98.78	5.28
Finland	2.83	0.00	0.00	2.09	1.23	0.24	29.48	4.17
France	1.58	1.53	7.42	1.79	3.08	0.60	1,701.16	0.18
Germany	10.63	9.92	11.21	14.45	11.55	2.27	1,751.11	0.66
Greece	0.21	0.29	2.27	0.26	0.76	0.15	22.38	3.40
Ireland	5.86	4.17	4.96	11.94	6.73	1.32	115.32	5.84
Italy	4.85	2.15	0.51	0.00	1.88	0.37	583.61	0.32
Japan	8.12	15.26	10.67	11.90	11.49	2.26	1,935.91	0.59
Luxembourg	0.16	0.20	0.15	0.54	0.26	0.05	11.53	2.25
Netherlands	5.67	0.03	4.99	2.38	3.27	0.64	480.16	0.68
New Zealand	0.49	0.32	0.58	0.20	0.40	0.08	20.71	1.93
Norway	14.14	14.89	0.85	13.88	10.94	2.15	388.52	2.82
Portugal	0.49	0.65	0.83	0.73	0.68	0.13	263.58	0.26
Spain	6.80	5.76	7.88	9.72	7.54	1.48	183.58	4.11
Sweden	9.14	10.66	11.65	10.45	10.48	2.06	347.66	3.01
Switzerland	1.68	1.96	1.30	1.45	1.60	0.31	166.63	0.96
United Kingdom	26.96	31.85	43.53	52.12	38.62	7.58	1,242.45	3.11
USA	102.13	141.31	306.97	303.91	213.58	41.94	8,687.63	2.46
**Multilateral**								
AfDF^a^	0.00	0.51	0.00	0.00	0.13	0.03	0.13	100.00
EC	9.79	17.61	30.33	39.11	24.21	4.75	970.94	2.49
GFATM	36.05	48.41	80.67	79.10	61.06	11.99	149.07	40.96
IDA^a^	13.37	39.08	54.64	0.00	26.77	5.26	26.77	100.00
UNAIDS	4.47	0.99	5.46	0.00	2.73	0.54	2.73	100.00
UNFPA	44.91	22.94	0.00	0.00	16.96	3.33	16.96	100.00
UNHCR^b^	1.16	1.68	0.66	1.25	1.19	0.23	61.23	1.94
UNICEF	21.00	17.22	19.53	15.93	18.42	3.62	132.24	13.93
UNOCHA^b^	0.02	0.00	0.74	0.42	0.30	0.06	4.59	6.54
WFP^b^	5.74	0.78	17.10	31.62	13.81	2.71	18.42	74.97
WHO^b^	0.33	0.11	0.80	0.50	0.44	0.09	1.75	25.14
**Total**	**354.17**	**409.81**	**643.12**	**630.05**	**509.29**	**100.00**	**20,794.54**	**2.45**

Only data from donors that disbursed ODA in 2003, 2004, 2005, 2006 presented.

a Disbursed funds were all for HIV/AIDS and STD control.

b Data from FTS (all other data from CRS).

Abbreviations: RH, Reproductive health; AfDF, African Development Fund; EC, European Commission; GFATM, Global Fund to fight AIDS, Tuberculosis, and Malaria; IDA, International Development Association; UNFPA, United Nations Population Fund; UNHCR, United Nations High Commissioner for Refugees; UNICEF, United Nations Children's Fund.

## Discussion

This study is, to our knowledge, the first systematic attempt to track ODA disbursement for reproductive health in conflict-affected countries based upon the CRS database. The results show that an average of $20.8 billion in total ODA was disbursed annually to the 18 conflict-affected countries included in this study between 2003 and 2006. Of this, an annual average of US$509.3 million (2.4%) was allocated to reproductive health. This amount represents an average of US$1.30 disbursed per capita per year in the 18 conflict-affected countries for reproductive health activities. No data exist on total actual reproductive health needs and the associated funding required specifically in conflict-affected countries. However, it is estimated that a total of $35.8 billion is required annually to meet reproductive health needs in all developing countries by 2015 [8]. The findings from this study provide evidence to suggest there is a substantial funding gap for countries affected by conflict, particularly as these countries generally have amongst the worst reproductive health indicators globally. More systematic and accurate information on the reproductive health needs in conflict-affected countries is required to better understand their funding requirements.

The study also shows that nonconflict-affected least-developed countries received 53.3% more reproductive health ODA per capita than the conflict-affected least-developed countries (Table 2), despite conflict-affected countries generally having greater reproductive health needs with the exception of HIV/AIDS (Tables 3 and 4). The findings demonstrate funding inequities between conflict-affected countries, as countries such as Democratic Republic of Congo and Somalia with the worse health and development indicators do not necessarily receive aid that is proportionate to need (Table 3). This finding supports those from previous studies, which estimate that conflict-affected countries have received 43% less overall aid than they should have received according to their levels of poverty [32]. This study echoes other studies that have shown gaps between funding and health needs in low-income countries [20].

This apparent inequity in funding patterns for reproductive health to conflict-affected countries may be partially explained by a combination of geopolitical and historical ties between donors and aid-recipients [32],[33]. There may also be concerns over governance and security in the recipient countries. Issues of absorptive capacity in the recipient government institutions and country more broadly are also significant [34]. In many conflict-affected countries government capacity is very limited making aid delivery highly challenging in practice. ODA is more likely to be disbursed to countries where capacity, infrastructure, and systems enable effective service provision [35]. The constraints of conflict-affected countries also mean that tangible returns on ODA may take longer to materialise, which may deter donor support [36].

Despite donor concerns with disbursing aid to conflict-affected countries, the data from this study suggest that donors are willing to increasingly engage in conflict-affected countries, with ODA increasing to the conflict-affected countries during the study period, and also conflict-affected countries receiving more per capita total ODA than nonconflict-affected countries (Table 2) although still not necessarily proportionate to need. The literature also suggests that although donor motivations vary substantially, donors are recognising the needs in conflict-affected countries and that disengagement from conflict-affected countries would have serious humanitarian and development implications [37]. As a result, the major donors appear to be willing to disburse funds to conflict-affected countries [10],[38]–[41]. This disbursement may be to governments or to nongovernmental organisations and UN agencies where there are concerns over the capacity of the government to manage those funds; with donors using a selective, carefully sequenced increase in aid for projects and programs tailored to weaker governance contexts [42]. This study did not analyse recipient institutions of the ODA. Future studies should be conducted to investigate the distribution patterns of reproductive health ODA between recipient governments and nongovernment organisations and agencies.

This increasing engagement with conflict-affected countries is not mirrored in disbursal of reproductive health ODA to conflict-affected countries, particularly non-HIV reproductive health ODA. While ODA distributed for reproductive health did increase by 77.9% between 2003 and 2006, two-thirds of this was due to a substantial increase in funding for HIV/AIDS activities. The funding for direct reproductive health activities, *excluding* HIV/AIDS activities, fell by 35.9% between 2003 and 2006. Family planning activities represent only 1.7% of the average annual ODA distributed for reproductive health activities, and funding for family planning activities dropped significantly in 2005 and 2006. In the case of some donors, this reduction in family planning activities may be partly explained by classification of certain family planning services, such as condoms, under HIV activities; and possibly individual donor funding cycles resulting in uneven distribution of funding. Previous studies have also shown that funding for family planning activities has decreased significantly for low-income countries since 1995 [7],[43]. This is despite coverage gaps for family planning in low-income countries [44].

Potential explanations for the findings that ODA disbursal rates for reproductive health activities (particularly non-HIV activities) are less than those in comparable nonconflict-affected countries could include the following. First, reproductive health may be given low priority by donors involved in giving aid to conflict-affected countries, and demand may be lacking for funds for reproductive health activities by recipient governments and nongovernmental organisations and agencies. Studies have linked the decrease in donor funding for reproductive health to increasing conservativeness towards reproductive health by donors, particularly under the last Bush administration [5]. There may also be hostility to reproductive health services by local actors, including belligerents. Second, there exists a lack of information on reproductive health needs in conflict-affected countries, including on the impact and effectiveness of reproductive health-related activities, to help inform ODA supply and demand. Third, there is a lack of capacity to fund and implement reproductive health activities in conflict-affected countries. Fourth, the common use of short-term ODA funding cycles to conflict-affected countries, which do not support the longer-term, sometimes less immediately tangible and quantifiable benefits of improved reproductive health outcomes. Underlying these possible causes is a lack of awareness of the low reproductive health funding for conflict-affected countries, a situation which this study seeks to help remedy.

In-depth, country-specific research is required to investigate the supply and demand characteristics of reproductive health ODA, including specific activities such as family planning. This should include the degree to which reproductive health activities are included in pooled humanitarian funding mechanisms, such as Common Humanitarian Funds and the Central Emergency Response Fund, and disbursements for reproductive health activities from philanthropic donors. Further investigation is also required to compare reproductive health outcomes between conflict- and nonconflict-affected regions. The findings from such studies should help inform initiatives to improve donor accountability and coordination, and ensure more equitable distribution of ODA to meet the reproductive health needs of populations affected by conflict [42],[45].

### Limitations

This study has a number of limitations. It explores only ODA, rather than national government expenditure on health from national revenues. It also does not include out-of-pocket expenditure on health [46]. Results from national-level studies on out-of-pocket expenditures on specific health activities could be combined with results from global-level tracking studies in future.

This study considered ODA disbursements rather than actual expenditure within countries. The distinction is important as there may be delays between the time aid is disbursed to a country and the time it is actually spent within a country. This delay may be due to bureaucracy, poor governance, or corruption. Outbreaks of violence might also pose a further challenge for both aid distribution and expenditure in conflict-affected countries. Although researching National Health Accounts and Health Sector Public Expenditure Reviews might provide the most comprehensive mechanism to track health sector financial resources at the national level, this activity remains logistically and methodologically challenging in several of the conflict-affected countries, given the inherent security, governance, and institutional weaknesses.

The study also relied on point estimates for the indirect reproductive health activities. A sensitivity analysis could have been conducted on the range of these estimates. However, limited data exist on the potential range of estimates and so was not considered to be meaningful. Further investigation of these ranges in conflict-affected countries would make a useful contribution to tracking reproductive health ODA.

This study highlights some limitations with the reporting of ODA for reproductive health using the CRS database. The use of CRS purpose codes for reproductive health presents limitations for assessing donor disbursements for specific sub-sectors of reproductive health—most noticeably for sexual and gender-based violence (SGBV) [47]. SGBV does not have a CRS purpose-code and, instead, activities related to SGBV are included in larger projects under human rights activities, elections, and postconflict peace-building activities. A separate purpose code for SGBV activities would enhance research tracking, effective policy analysis and decision-making for resource allocation on SGBV.

Other limitations concerning CRS reporting structures relate to countries in which conflict is limited to a specific geographic area, such as in northern Uganda. Using CRS, it is not possible to reliably determine the beneficiaries of the ODA and therefore not possible to get an accurate picture of the extent to which ODA is disbursed to the most conflict-affected populations (e.g., internally displaced persons in northern Uganda).

CRS may not capture all governmental donor aid flows. A number of governments provide ODA but do not report to CRS, such as China, Russia, and Saudi Arabia; some regional development funds may also not report to CRS. Regional and multi-country disbursements of ODA were also not included in the analysis because they could not be disaggregated by country. ODA between developing countries is also not included in CRS. However, it is estimated that the CRS covers approximately 90% of all ODA to developing countries, and its use has been justified in other published studies on ODA tracking [20],[21].

Significantly, the CRS also does not include direct aid disbursements by philanthropic organisations. This omission is principally because publicly available data for aid disbursements from major philanthropic organisations such as the Bill and Melinda Gates Foundation are often incomplete and not standardised, because philanthropic organisations are not required to report funding in the same way as institutional donors are [29]. These organisations may provide between 8% and 15% of funding for HIV/AIDS and reproductive health activities in low-income countries [48]–[50]. However, the available literature suggests that most of the funding from the Bill and Melinda Gates Foundation appears to be allocated to research institutions, universities, and civil society institutions in high-income countries (although with end beneficiaries predominantly in low-income countries) [29],[48]. Reliable evidence could not be found in the literature on the impact of the Bill and Melinda Gates Foundation or other private organizations on aid to conflict-affected countries. More systematic and standardised financial reporting by philanthropic organisations would significantly support aid tracking, particularly if their reporting could be harmonised with the CRS.

There are also a number of administrative limitations with the CRS reporting system. First, it relies on data from the existing financial systems of donor organizations. Some donors may have different classification systems, and linking these to the CRS system is a challenge [51]. Second, problems in the completeness of data for the DAC member countries have been noted [22]. Donors might not be accurately reporting ODA data to CRS, and/or CRS might not be accurately recording the data. There may also be misclassification of disbursements. For example, a donor may classify family planning activities as reproductive health activities. Third, the CRS database was not originally designed to be analysed for specific sectors such as health, so details on these sectors can be limited or difficult to access [22]. This problem is reflected in the fact that a project that supports more than one sector is categorized according to the sector receiving the majority of funds, and the other sectors are classified as receiving no funds from that particular project. Fourth, descriptive information on projects, which is important for determining the precise nature and purpose of any aid transaction, is often missing. There are also gaps for the multilateral institutions that report to the CRS on a voluntary basis, as they are not DAC members.

The FTS was used to obtain data for institutional ODA disbursements from UNHCR, UNOCHA, WFP, and WHO. The amounts of reproductive health ODA reported by these four institutions through FTS was low compared to that reported by other donors in CRS (3.1% of all reproductive health ODA disbursed). This low amount could be explained by the fact that the four institutions are reporting only institutional disbursements, rather than ODA provided by other multilateral and bilateral donors for country-specific activities by these four agencies, which is reported to CRS under these other multilateral and bilateral donors. In the case of UNHCR, many of their health activities are implemented by nongovernmental agencies, which may receive separate funding from other bilateral and multilateral donors. In addition, most of UNHCR's activities are for refugees in countries neighbouring the conflict-affected countries (although this is now changing due to UNHCR's increasing responsibility for internally displaced persons as a result of reforms in late 2005) [52]. An alternative source of information for these four UN agencies could have been their annual reports and financial statements, but there are several limitations to this approach. First, the reports and statements did not all report by calendar year so the data could not be synchronised with the time-scale used for this study. Second, the reports and statements did not all specify which activities their funds were for (e.g., health or food or education or other unrelated sectors). Third, it was not clear if the financial statements included funds received from bilateral and multilateral agencies that were also recorded in CRS, which could have resulted in double counting.

The study was limited to 4 years. Additional years would help to provide a longer-term trend analysis of ODA to conflict-affected countries, and a follow-up study is planned to track ODA disbursal for reproductive health up to at least 2010.

### Conclusion

Studies have observed that progress towards the Millennium Development Goals in conflict-affected countries is generally slower than in nonconflict-affected countries and that substantially more resources need to be mobilised and better spent in conflict-affected countries [9],[32],[38]. This is the first study to our knowledge to provide evidence of inequity in disbursement of reproductive health ODA between conflict-affected countries, and in comparison with nonconflict-affected countries. It explicitly demonstrates declining funding for non-HIV reproductive health activities in conflict-affected countries. The study highlights the need for future research to strengthen understanding on funding for reproductive health activities in conflict-affected countries, and to influence policy makers and support initiatives to make aid financing more responsive to need [42],[53].

## Abbreviations

CRS: Creditor Reporting System
DAC: Development Assistance Committee
FTS: Financial Tracking System
MDG: Millennium Development Goal
ODA: official development assistance
OECD: Organisation of Economic Cooperation and Development
SGBV: sexual and gender-based violence
UNHCR: United Nations High Commissioner for Refugees
UNOCHA: United Nations Office for the Coordination of Humanitarian Affairs
WFP: World Food Programme
WHO: World Health Organization
